# Effect of dietary pumpkin (*Cucurbita moschata*) seed meal on layer performance and egg quality characteristics

**DOI:** 10.5713/ab.21.0044

**Published:** 2021-06-24

**Authors:** Petru Alexandru Vlaicu, Tatiana Dumitra Panaite

**Affiliations:** 1 Department of Chemistry and Animal Nutrition Physiology, National Research and Development Institute for Animal Biology and Nutrition, Balotesti, Ilfov, 077015, Romania

**Keywords:** *Cucurbita Moschata*, Egg Cholesterol, Egg Fatty Acids, Egg Quality, Storage Time

## Abstract

**Objective:**

The objective of this study was to investigate the effect of dietary pumpkin (*Cucurbita moschata*) seed meal (PSM) on laying hens’ performance, quality, fatty acids, cholesterol, antioxidant compounds and shelf life of eggs.

**Methods:**

Eighty Tetra SL laying hens, 50-week-old, were randomly divided into two equal groups, having 10 replicates with 4 birds in each. The control (CON) treatment was fed with basal diet, while experimental treatment was fed a diet with 9% PSM, for a 6 week period.

**Results:**

Dietary PSM significantly decreased average daily feed intake (p<0.05), with no significant effect on other performance parameters. The PSM, enriched the eggs with polyunsaturated fatty acids, especially α linolenic acid (0.33 vs 0.21 g/100 g) and linoleic acid (20.65 vs 18.37 g/100 g), whereas it reduced the amount of arachidonic acid with 3.91% and n−6/n−3 ratio in PSM eggs compared with CON. The inclusion of 9% PSM significantly (p<0.05) diminished the cholesterol concentration in yolk with 11.31% and in egg with 10.38%, in respect to the CON samples. The significantly (p<0.05) higher concentration of polyphenols and antioxidant compounds, determined in PSM eggs, proved to be effective on shelf life of eggs preserved at refrigerator (5°C) and room temperature (21°C) for 28 days, by delaying the lipid oxidation and protein denaturation. This effect was reflected in significantly (p<0.05) higher Haugh unit in eggs stored 28 days at 21°C and lower albumen pH values for the overall storage time, both at 5°C and 21°C, proving the antioxidant effect of pumpkin.

**Conclusion:**

Dietary PSM supplementation was significantly effective on average daily feed intake and egg quality by increasing some fatty acids while lowering the cholesterol concentration. Also, PSM proved to be effective improving shelf life of eggs for 28 days storage time.

## INTRODUCTION

The egg has great nutritional value, because it is rich in proteins, fats, fat-soluble vitamins, and minerals. Its inclusion in the daily diet is important for human health. Despite the nutritive quality of the egg, during the last decades, the potential association with cardiovascular disease related to the harmful effect of cholesterol and saturated fatty acids (SFA), raised concerns against frequent egg consumption [1,2]. Egg is also a cheap food with high nutritional value that contributes to cover the nutritional requirements of several human societies [3]. Nonetheless, half a century ago it was criticized due to its cholesterol content (50 g egg contains 186 mg cholesterol) thus exceeding by 50% the daily intake. It is difficult to modify egg cholesterol content, however, its lipid profile can be modified through hen feeding with oil seeds meals such as flaxseed, canola, cotton seed or rapeseed by reducing the content in n−6 fatty acid (FA) whereas increasing n−3 FA [4,5]. Using diets rich in polyunsaturated fatty acids (PUFA) to increase egg yolk FA content, while trying to decrease the egg cholesterol concentration requires adding an antioxidant (natural or synthetic) to prevent early lipid oxidation of eggs. Moreover, there is a need to extend shelf life of eggs in different storage conditions, especially if the eggs are rich in fats to prevent albumen protein oxidation. Such candidate that can be used as natural source of PUFA and antioxidant compounds could be the waste of pumpkin seed oil extraction, as meal. According to Valdez-Arjona et al [6], global pumpkin production in 2017 was more than 27 million tons distributed across all continents. The wastes resulted from these tons if discarded in the soil could affect the human and some animals’ health through soil pollution. Pumpkins used in animal feed could improve the quality of eggs, meat or milk due to their moderate to high content of antioxidants (lutein, phytosterols, α-tocopherol and β-carotene) and PUFAs, depending on which type of *Cucurbita* is used [7]. Literature data search revealed varied reports that described the chemical composition and pharmacological properties of pumpkin by-products, whereas the largest number of studies regarding the use of pumpkin by-products as animal feed ingredient has been conducted in broiler chickens and pigs [8,9]. Hajati et al [10] showed that addition of 5 g/kg diet of pumpkin seed oil to laying hens diets does not affect the productive performance, while no changes in the laying rate or the egg quality of laying hens fed with pumpkin seed flour were observed by others [8,11]. Furthermore, Martínez et al [12] reported that 10% of *Cucurbita maxima* seed meal in laying hens’ diet, increased more than double (from 454 mg/100 g to 1,095 mg/100 g of yolk) the content n−3 FA, while obtaining a 10% cholesterol content decrease in the egg. However, very limited information has been reported in the literature on the dietary effect of pumpkin seed meal especially *Cucurbita moschata* and its possible effect on hens’ performance and egg quality. With this regard, the purpose of this study therefore was to investigate that if 9% pumpkin (*Cucurbita moschata*) seed meal can be added to the diets without altering the laying performance and the diverse effect on internal and external egg qualities, including FA composition, cholesterol concentration, antioxidant compounds and shelf life.

## MATERIALS AND METHODS

### Experimental procedure

The experimental procedures complied with the Romanian legislation (Law 206/2004, ordinance 28/31.08.2011, Law 43/11.04.2014, Directive 2010/63/EU) according to an experimental protocol approved by the Ethics Commission regarding the progress of the experiments from our institution.

### Animals, experimental design and diets formulation

The experiment was conducted for six weeks (42 days). In total, eighty, 50-week-old Tetra SL layers were randomly distributed into two groups (control treatment [CON] and pumpkin seed meal [PSM]), with 40 layers each having 10 repetitions of 4 hens per pen in each group. The hens were housed (four hens/cage) in an experimental hall equipped with Zucammi three-tier digestibility cages (according to sanitary - veterinary recommendations) and controlled microclimate conditions (temperature: 20°C to 22°C, humidity 65% to 68%), which allowed recording daily feed intake and egg production. The lighting regimen (16 h:8 h) and birds handling were applied according to the Management Breeding Guide.

Diets were formulated according to the results of the chemical analysis of the feed ingredient, and supplied the layers’ nutritional requirements, considering the hybrid, age, and the nutritional requirements of the Tetra SL (LL-Hybrid) hens. Table 1 shows the composition of both diets. The control diet (CON) consisted mainly of corn, soybean meal and corn gluten, containing 16.50% crude protein (CP) and 2,750 metabolizable energy (Kcal/kg). The difference between the CON and PSM treatment was given by the supplementation with 9% pumpkin seed meal. The access to feed and water was provided *ad libitum*.

### Laying performance measurements

The following performance parameters were monitored during the entire experimental period: average daily feed intake (ADFI, g/d/layer), feed conversion ratio (g feed/g egg mass), laying percentage (%), and average egg weight (g/d). The egg production was recorded daily. Feed conversion ratio (FCR) was calculated by measuring the feed consumption and egg weights produced daily.

### Chemical analysis

The basic chemical composition analyses were determined on samples dried at 65°C, as previously described [13]. Briefly, standardized methods were used to determine the nutrient concentration. The CP was determined by the Kjeldahl method according to Regulation (CE) nr. 152/2009 and standard SR EN ISO 5983-2:2009 (Kjeltec auto 1030; Tecator Instruments, Hoganas, Sweden). Crude fat (EE) was determined by extraction in organic solvents - the method complies with Regulation (CE) No. 152/2009 and standard SR EN ISO 6492:2001 (Soxtec 2055; Foss Tecator, Hoganas, Sweden). Crude fibre was determined by the method with intermediary filtration, according to Regulation (CE) No. 152/2009 and standard SR EN ISO 6865:2002 (Fibertec 2010 System; Foss Tecator, Sweden). All assays were performed in triplicate.

### Sample collection and procedures

Samples of compound feeds (about 500 g from each manufactured diet/group) were collected for the basic chemical composition, FA composition and antioxidant compounds.

At the end of the experiment, 18 eggs/group were collected and weighed (g) in a precision scale and sent to the laboratory, to determine the external and internal egg quality parameters as follows: albumen weight (g), yolk weight (g), shell weight (g), eggshell thickness (mm), eggshell breaking strength (kf), yolk color intensity, albumen pH, yolk pH and Haugh units (HU) were measured. After these measurements, yolk samples were used for determination of yolk FAs, fat content, total antioxidant compounds and egg cholesterol concentration.

Further, to determine the effect of PSM on eggs shelf-life freshness parameters, we collected a total of 30 eggs from each group, and after we divided them in five. Each of 6 sampled eggs was stored in chambers for 14 and 28 days respectively, in a refrigerator (5°C) and at room temperature (21°C). For sampling, each egg was weighed and broken, the white and yolk were separated and in each egg the pH of the white and yolk and the HU were determined.

### Determination of external and internal egg quality analysis

Egg, albumen, yolk, and eggshell weight measurement were made with a Kerm scale (0.001 precision), yolk color intensity and HU were determined using the Egg Analyzer TM, (type 05-UM-001), manufactured by Orka Technology Ltd; eggshell thickness and eggshell breaking strength with an egg force reader device, (Sanovo engineering A/S; Odense, Denmark); white and yolk pH with and portable pH meter (Five Go F2-Food kit with LED 427IP67 Sensor Mettler Toledo, Odense, Denmark).

### Determination of antioxidant compounds

The total polyphenol content (TPC) in the methanolic extracts of the diets and eggs was determined spectrophotometrically, using Folin-Ciocalteu’s reagent. Briefly, 0.5 mL of the thyme extract was added into a 50 mL volumetric flask containing 2.5 mL of Folin-Ciocalteu’s reagent, 30 mL of distilled water and 7.5 mL of 20% Na_2_CO_3_ and filled up to the mark with distilled water. Two hours later, the absorbance of blue coloration was measured at 765 nm against a blank sample. Gallic acid was used as the standard and the results are expressed as mg/L of gallic acid equivalents/g sample (mg GAE/g). The flavonoid content of the methanolic feed extracts was determined according to the method described by Zhishen et al [14], and the results are expressed as mg rutin equivalents/g sample (mg Rutin/g). Total antioxidant capacity (TAC) of the methanolic feed, albumen and yolk egg extracts was determined by the diphenylpicrylhydrazyl radical (DPPH) method proposed by Marxen et al [15]. The results were expressed as mM trolox/sample (mM Trolox).

### Determination of yolk fatty acids

The FA content of pumpkin seed meal, feeds and egg samples was determined using a gas chromatograph PerkinElmer Clarus 500 (Waltham, MA, USA), as previously described elsewhere by Turcu et al [16]. The principle of the method consists of the transformation of FAs, from the sample under analysis, into methyl esters, followed by the separation of the components on the chromatographic column, their identification being made by reference to the standard chromatograms. The chromatograph has a flame ionization detector (FID) and capillary separation column with a high polar stationary phase TRACE TR-Fame, (Thermo Electron, Waltham, MA, USA), with dimensions of 60 m×0.25 mm×0.25 μm film. The average amount of each FA was used to calculate the sum of the total SFA, monounsaturated fatty acids (MUFA), and PUFA.

### Determination of cholesterol concentration in egg

Cholesterol was determined from samples dried at 65°C and performed using a gas chromatographic (GC) method in accordance with AOAC Official Method International 1996 99410. The method involves saponification of the sample by reflux boiling in a solution of methanol and potassium hydroxide (5% KOH in methanol), followed by extraction in petrol ether, concentration in rotavapor, and addition of chloroform, followed by extraction in petroleum ether and pouring on chloroform after concentration. The sample was split in GC (Perkin Elmer Clarus-500; FID), separated by chromatography column (HP-5 capillary 30 m, 0.3 mm ID, 0.1 μm. df thick film) and compared with standard chromatograms by measuring the peak area.

### Statistical analysis

The effects of PSM on laying hens’ performances and egg quality were analyzed by one-way variance analysis using StatView for Windows (SAS, version 6.0). The results were expressed as mean values. The differences among treatments were considered significant at p<0.05.

## RESULTS

### Proximate chemical composition of dietary pumpkin seed meal

The proximate chemical composition of the dietary PSM (*Cucurbita moschata*) is presented in Table 2. From our results, it was observed that PSM is a rich source of protein and fat, presenting high concentration of PUFA and antioxidant compounds (polyphenols, antioxidant capacity, and flavonoids). It must be noted that the nutritional composition of pumpkins is variable and depends on several factors, like growing conditions and the species.

### Laying hens performances and quality characteristics of eggs

The effect of dietary PSM on laying hens’ performances were calculated three times, as presented in Table 3. From the obtained results, only ADFI production parameter was significantly (p<0.05) lower in PSM group compared with CON group. A tendency of higher laying rate and FCR in CON treatment was observed for the overall period (42 days).

The influence of PSM on internal and external egg quality characteristics, from the analyzed eggs, is presented in Table 4. It was observed that neither egg weight nor egg constituents were affected by the inclusion of PSM in diet. There was only a tendency of increasing albumen weight, shell strength, yolk color and HU in PSM samples, while yolk weight and albumen pH tended to be higher in CON samples, but the differences were not statistically sustained (p>0.05).

### Effect of dietary pumpkin seed meal on egg yolk fatty acids and cholesterol

Table 5 shows the FAs profile in the egg yolks from laying hens fed up to 9% PSM in comparison with a CON diet. Myristic acid (C14:0), palmitic acid (C16:0), and stearic acid (C18:0) are the most representative SFA in the yolks, from both CON and PSM treatments. It was observed that myristic acid (C14:0) was significantly (p<0.05) higher in CON samples while heptadecanoic acid (C17:0) was significantly higher (p<0.05) in PSM egg yolk samples. From the total of MUFA, the oleic acid (C18:1), was the most abundant which tended to be higher in the CON egg samples, compared with PSM egg samples. Among the PUFA, the n−6 FA, linoleic acid (C18:2n−6) was predominant (p< 0.05) in the yolks from PSM compared with CON egg yolk samples. The same occurred with the n−3 PUFA α-linolenic acid (C18:3n−3). Both showed significant differences (p< 0.05) compared with the CON eggs, being with 11.04% respectively 36.36% higher. Besides, the arachidonic acid (C20:4n6) decreased in respect to the inclusion level of PSM, while the precursor of α-linolenic acid (C18:3n−3), the docosahexaenoic acid (C22:6n3), increased significantly (p<0.05) in PSM egg yolk samples. The n6/n3 ratio, was also significantly (p<0.05) lower in PSM group compared with CON.

The total cholesterol in the egg and egg yolk from laying hens was superior (p<0.05) for the CON samples compared with PSM samples. The egg yolk cholesterol was reduced significantly (p<0.05) when including the PSM in the diets (Table 5). The reduction of cholesterol in yolk was a positive effect, considering the several diseases assumed to be caused by the excessive intake of this lipid.

### Effect of pumpkin seed meal on total antioxidant compounds in eggs

**Figure 1:**
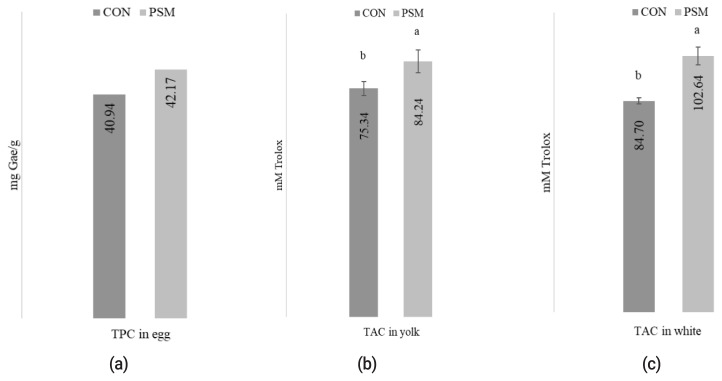
The effect of dietary PSM on (a) TPC mg GAE/g, (b) TAC from yolk, and (c) white egg (mM Trolox) in comparison with control (CON) diet. PSM, pumpkin seed meal; TPC, total polyphenol content; CON, control; GAE, gallic acid equivalents. ^a,b^ Bars with different superscript letter are different (p<0.05).

The effect of PSM in comparison with CON on the TAC determined in albumen and yolk egg and TPC in eggs is presented in Figure 1 (a, b, c). The oxidative status of TPC in methanolic egg extracts was significantly (p<0.05) higher in PSM egg samples compared with CON. Also, the methanolic extracts, determined in yolk (82.24 mM Trolox/g) and albumen (102.64 mM Trolox/g) exhibited statistically significant (p<0.05) differences in TAC, as measured using the DPPH method.

### Changes of egg quality with storage time and temperature

**Figure 2:**
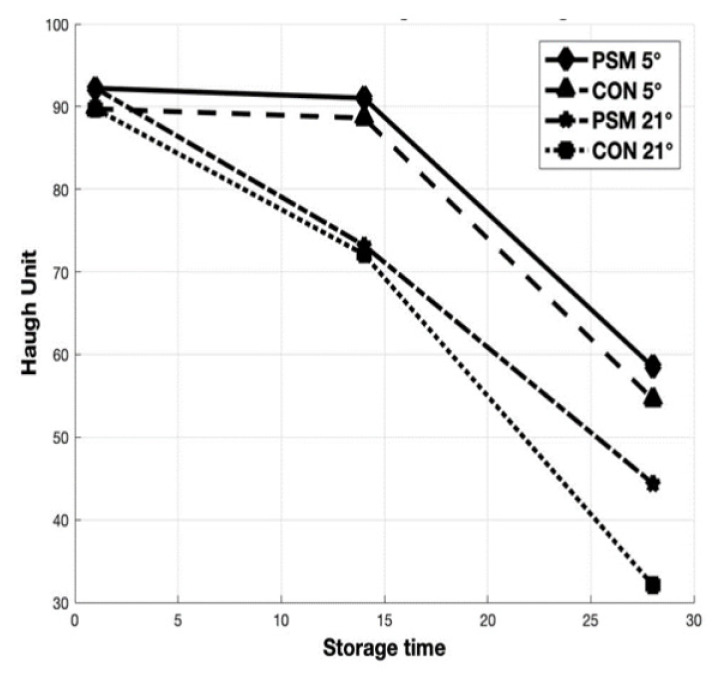
Relationship between storage time and HU from CON and PSM eggs stored for 14 and 28 days at 5°C and 21°C. HU, Haugh unit; CON, control; PSM, pumpkin seed meal.

**Figure 3:**
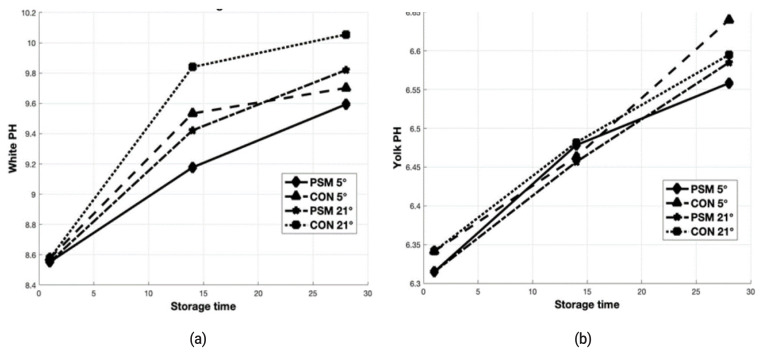
Comparison of CON vs PSM on (a) white pH and (b) yolk pH evolution of eggs stored for 14 and 28 days at 5°C and 21°C. CON, control; PSM, pumpkin seed meal.

The results of the storage time and temperature effects on freshness indicators as HU, albumen and yolk pH are presented in Figure 2 and Figure 3 (a, b). Storage time and temperature significantly (p<0.05) affected the freshness parameters investigated in the present study after 14 and 28 days of storage at 5°C and 21°C. The most visible decrease of HU was observed for CON eggs stored at 21°C for 28 days. Regarding the albumen pH, as a response to dietary PSM (Figure 3A) was significantly (p<0.05) lower in eggs stored 14 days, both at refrigerator (9.18 vs 9.53) and room temperature (9.42 vs 9.84) compared with CON. The albumen pH was not significantly (p>0.05) affected by storage time for 28 days at 5°C, but those from PSM stored at 21°C, had a significantly (p<0.05) lower pH (9.82) compared with CON samples (10.06). The yolk pH value significantly (p<0.05) increased with increasing storage period (Figure 3B). High storage temperature also increased the yolk pH after 28 days storage at 5°C and 21°C. Although the pH of the egg albumen and yolk increased linearly along with the storage time and temperature, the changes of yolk pH were not as large as those from albumen pH.

## DISCUSSION

### Proximate chemical composition of dietary pumpkin seed meal

Most of the PSM nutrient composition was in range with previously reported results [12,17]. Kim et al [17], evaluated three species of pumpkin (*Cucurbita pepo*, *Cucurbita moschata*, and *Cucurbita maxima*) and reported that *Cucurbita maxima* contains significantly (p<0.05) more carbohydrates, fat, and fiber, compared with *Cucurbita pepo* and *Cucurbita moschata* which have higher protein content (29.81%) in the seeds, being 3% higher than we found in our study (Table 2). Regarding the crude fiber content in PSM and diet, Wafar et al [18] determined similar concentration (21.58%) when PSM (*Cucurbita pepo*), up to 20% was used in broilers diet. Others, [12,17], found higher crude fiber (27.54% and 34.41%) and CP (34.88% and 37.91%) concertation, with close values in terms of PUFA from PSM. Contrary, Martínez et al [8] determined lower PUFA concentration (15.23 g/100 g PUFA) in PSM (*Curcubita maxima*), which is a low concentration compared with our results (52.19 g/100 g PUFA). The content of the polyphenols, antioxidants and flavonoids in pumpkins has also been studied and described [19,20] previously, and it was reported that *Cucurbita moschata* contains numerous phenolic compounds, phenolic acids (tyrosol, vanillic acid, vanillin, luteolin, and sinaptic acid), flavonols (rutin, kaempferol, isoquercetin, astragalin, myricetin, and quercetin), oxalate and sterols (stigmastatrienol and spinasterol). These differences in terms of chemical composition of PSM depend on both the species and the variety of pumpkin. Also, factors like the soil aeration, soil moisture, soil pH, climate, growing conditions, harvesting, deposition, temperature, and light intensity. Besides all these natural influential factors, processing methods also have a significant effect on the proximate chemical composition of pumpkin meal [21].

### Laying hens performances and quality characteristics of eggs

Despite of the great nutritional value of PSM, there are few data in the literature on the effects of the dietary inclusion of *Cucurbita moschata* on production performances in hens. The largest numbers of studies regarding the use of PSM as animal feed have been conducted in broiler chickens and pigs in which an increase in weight gain was observed [9,18]. In laying hens, it was reported [8] that including four levels of pumpkin seed meal, had no effect on productive performances. Similarly, it was shown [22,23] that the neither the addition of pumpkin oil nor fluted pumpkin (*Telfaria occidentalis*) leaf extract to laying hens diet had any effect on productive performances. These results are also sustained by Chiroque et al [24] who assumed, as in our case, that crude fiber content is responsible for influencing feed intake results. From the current available data, on the influence of PSM on fresh egg characteristics, it can be assumed that 9% PSM does not affect the internal or external quality parameters of the eggs. Overall, the PSM diet was not a significant (p>0.05) contributor regarding the internal and external egg quality characteristics or the obtained performances, excepting ADFI.

### Effect of dietary pumpkin seed meal on egg fatty acids and cholesterol

In our study, manipulating laying hens’ diet by adding pumpkin seed meal resulted in significant changes in the FA profile of eggs. From the determined egg yolk FA, α-linolenic acid (C18:3n−3) was significantly (p<0.05) increased in PSM eggs compared with CON samples, while decreasing the arachidonic acid (C18:4n−6). Martínez et al [8] confirmed same decline in arachidonic acid when including up to 10% PSM (*Cucurbita maxima*), in layers diets. This result is wanted, because PUFA (n−3 and n−6) are essential for human consumption. They provide healthy growth and development and play an important role in the prevention of some disease and can modulate lipid metabolism in a beneficial way [25]. Overall, the total SFA, MUFA, and n−6 PUFA, had close values between the two groups, while the total PUFA and n−3 PUFA were significantly (p<0.05) increased in response to the 9% PSM addition to laying hens’ diet. The important ratio of n−6/n−3 was lowered (p<0.05) with 15.52% in PSM eggs, compared with the CON eggs. Both n−3 PUFA and n−6 PUFA together with their ratio are the principal FA controlling the hypocholesterolemic index in eggs. The n−3 PUFAs plays a major role for regulating the thrombogenicity indices, whereas n−6 PUFAs are dominant for the atherogenicity indices [26]. Pumpkin seeds fall into the small and privileged group of oilseeds with essential FA, although variability in n−3 and n−6 should be considered, depending on the species and varieties [13]. The eggs of the hens from PSM treatment, were enriched in approximately 120 mg/100 g of yolk of α-linolenic (C18:3n−3) compared with the CON eggs. Previously, [9] also obtained positive effects in the content of n−3 PUFA in the egg yolks, when supplementing the hens’ diets with 10% PSM. This proves that the level of inclusion of n−6 in the diets affects the percentage incorporated in the egg. In the studies and clinical trials from literature on the introduction of feeds rich in α-linolenic FA (C18:3n−3) as meals, wastes, oils, vegetable by-products, in diets of laying hens proved there is trend toward the enrichment of the egg with the n−3 FA [25]. The sum of MUFA decreased while the PUFA in the egg yolk was increased as a response of 9% inclusion level of PSM, due to the concentration of the oleic (C18:1), linoleic (C18:2n6) and α-linolenic (C18:3n3) FA in the PSM, and to the serum circulation and its incorporation to the egg [7]. According to Grobas et al [5], due to the physiological peculiarities of the birds, the incorporation of the FA resulting from elongation and desaturation could be poor sometimes. Poultry feed manipulation can be used as a successfully tool to increase the amount of n−3 directly, by feeding meals or other vegetable by-products rich in fat. Increasing the PUFA in eggs could contribute to the higher dietary intake of n−3 PUFA as an alternative source of n−3 FA. Hence, using diets rich in n−3 FA reduces the n−6 FA contents of egg yolk, which represent a favorable effect for consumers. From a nutritional point of view, as it was shown by Kouba et al [27] the PUFA content in eggs can significantly influence human health providing beneficial effects. The n−3 FA can be efficiently passed along to the human food chain with the practice of using diets rich in PUFA with or without antioxidants in poultry feeding.

The two common expressions of egg cholesterol concentration (g cholesterol/g yolk and g cholesterol/g egg) exhibited opposite trends when plotted against the CON samples (Table 5). The cholesterol reduction in the yolk and egg could be determined by the presence of unsaturated fatty acids and dietary fiber in the PSM (21.11%) or by the presence of phytosterols (campesterol and beta-sitosterol) [28]. The hypocholesteric impact of PSM in decreasing the cholesterol concentration in this study is consistent with those obtained previously by others [29], in which the significant (p<0.05) interaction on egg and egg yolk cholesterol was confirmed, but inconsistent with Ceylan et al [30] who reported that different fat source supplementation resulted in differentiated level of cholesterol in the experimental groups. Similar reduction in egg cholesterol was observed previously [31], which proved the hypocholesterol effectiveness of the sterols in the pumpkin and its by-products. Nevertheless, despite the beneficial characteristics of the PSM, it was reported [31] that the egg yolk cholesterol was not decreased in a progressive form. By comparing these facts in can be concluded that in terms of lowering the cholesterol concentration, not only the dietary supplement or the added dose is important, but the correct FA profile is also an important factor. These results could contribute to improve the human diet since the consumption of cholesterol and some lipids is considered unhealthy.

### Effect of pumpkin seed meal on total antioxidant compounds in eggs

Supplementing laying hens’ diet with 9% PSM promotes the enrichment with antioxidant compounds in egg white and yolks. This is a beneficial result, because antioxidant compounds act synergistically with each other, providing a protective effect against eventual lipid peroxidation [32], especially in eggs with high PUFA concentration. For this reason, simultaneously enrichment of eggs with polyphenol and antioxidant compounds was suggested to decrease FA oxidation and provide a good source of dietary antioxidant [33] which have been associated with beneficial effect on human health. In line with our results, other author [34] proved the antioxidant effect of Asian pumpkin on eggs. Similarly, Meineri et al [35], used pumpkin seed (50 g/kg) as natural antioxidant to supplement a linseed broiler diet and obtained a significant decrease (p<0.05) of TBARS values from meat compared with groups without pumpkin. High antioxidant capacity in eggs was also reported when hens were fed with fat and antioxidant meals (8.672 mM equiv. vitamin C) in PUFA enriched eggs compared with control group [36]. In another study, it was reported that PUFA enriched eggs simultaneously with TPC and TAC help antioxidant assimilation [37], which can explain our results regarding the increase values of antioxidant compounds in eggs from PSM group. Studies reporting the total antioxidant compounds on layers eggs fed with different natural or synthetic antioxidant sources are common, but from our knowledge there is no data in literature, regarding the effect of pumpkin seed meal on delaying lipid oxidation of eggs.

### Changes of egg quality with storage time and temperature

The storage stability of preserved eggs over extended periods is of interest to manufacturers of packaged food. The HU is the most widely used measure to assess egg freshness. In our study, there were significant (p<0.05) differences between CON and PSM egg samples, but the most visible decrease in HU was observed for CON eggs stored at 21°C for 28 days (Figure 2). The HU for PSM samples stored for 28 days at refrigerator was with 6.66% higher, while the samples stored at room temperature were with 27.65% higher compared with CON. The HU decreased from initial value of 89.73 to 72.13 on day 14, and further to 32.12 on day 28 in CON samples at room temperature, while PSM samples from the initial 92.22 decreased to 73.22 respectively 44.40 during 28 days of storage, at room temperature (21°C). The same trend of decreasing in HU parameter was observed for samples stored at refrigerator (5°C) for 28 days. This decline was from 88.62 at day 14, to 54.62 at day 28 (CON) and from 91.03 to 58.52 (PSM), respectively. Haugh unit values decreased below 70 after 14 days storage in the current study, which concurs with the findings of others [38], but overall, HU was greater for PSM than CON eggs. Similarly, Akyurek and Okur [39] reported dramatic reductions in HU eggs (91.30 to 72.63) stored at 29°C for 10 days, whereas at 5°C no decline was found. Also, Dong et al [40], reported that storage time and temperature adversely affected HU (p<0.001).

The changes that occur in egg during storage are many and complex and affect the functional properties of white and yolk egg. These changes include thinning of albumen, increase of pH, weakening and stretching of the vitelline membrane and increase in water content of the yolk. Egg white pH is strongly correlated to the HU and is rarely affected by age of hens or strain, except for storage [38]. With respect to the effect PSM on storage time and temperature on the physiochemical properties of eggs, we observed significant (p<0.05) increases in both white and yolk pH with increasing storage time and temperature. Previously it was reported that the white and yolk pH from commercial eggs was significantly affected by storage period but not by temperature [39]. These changes are made by the significant physical changes occurred in the white viscosity with increasing temperature and storage period [40]. The deterioration in white and yolk quality was clearly pronounced during storage at 21°C for 28 days. Yolk pH increases because of absorption of water from the white or lipid peroxidation of PUFA, while the albumen pH increases due to protein deterioration [41]. Overall, the antioxidants and polyphenols from PSM, acted against egg oxidation, compared with CON, which is similar with recent finding of especially for samples stored at refrigerator for 28 days.

## CONCLUSION

Including up to 9% PSM in the diets of laying hens increased the total PUFAs, especially the beneficial FAs for human consumption and reduced the harmful ones. Additionally, the total cholesterol from egg and yolk was significantly reduced in the eggs, increasing the concentrations of total polyphenols and antioxidants in yolk and white egg. The interaction effects between storage period and temperature were significant for HU, yolk pH and white pH, as a response to dietary supplementation with pumpkin seed meal. Further experiments are needed to test different levels and their interactions on laying hens of this valuable source of nutrients.

## Figures and Tables

**Table 1:** Ingredients and calculated composition of diets

Items	CON	PSM
Ingredients (%)		
Corn	57.45	58.68
Soybean meal	21.24	16.50
Sunflower meal	7.00	5.00
Pumpkin meal	0.00	9.00
Sunflower vegetal oil	2.02	1.34
L-lysine-HCl	0.00	0.24
DL-Methionine	0.10	0.22
Choline	0.05	0.05
Calcium carbonate	9.91	9.52
Phosphate	0.86	0.85
Mycotoxin inhibitor	0.05	0.05
Sodium chloride	0.35	0.36
Premix^1)^	1.00	1.00
Total	100	100
Calculated nutritional composition		
Metabolizable energy (Kcal/kg)	2,750	2,750
Dry matter (%)	87.13	88.21
Crude protein (%)	16.50	16.50
Crude fat (%)	3.83	4.45
Crude fibre (%)	4.49	6.00
Determined nutrients		
Total PUFA (g/100 g total FA)	55.77	55.43
n−6 PUFA (g/100 g total FA)	54.20	52.93
n−3 PUFA (g/100 g total FA)	1.57	2.98
n−6/n−3 ratio	34.52	17.59
Polyphenols (mg/g)	16.10	18.89
Antioxidant capacity (mM Trolox)	7.60	9.22
Flavonoids (mg rutin/g)	6.47	6.83

CON, control diet; PSM, control diet supplemented with 9% pumpkin seed meal; PUFA, polyunsaturated fatty acids; FA, fatty acids.

1) The premix provided the following per kilogram of diet: vitamin A 13,500 IU; vitamin D_3_ 3,000 IU; vitamin E 27 mg; vitamin K_3_ 2 mg; vitamin B_1_ 2 mg; vitamin B_2_ 4.8 mg; pantothenic acid 14.85 mg; nicotinic acid 27 mg; vitamin B_6_ 3 mg; vitamin B_7_ 0.04 mg; vitamin B_9_ 1 mg; vitamin B_12_ 0.018 mg; vitamin C 25 mg; manganese 71.9 mg; iron 60 mg; copper 6 mg; zinc 60 mg; cobalt 0.5 mg; iodine 1.14 mg; selenium 0.18 mg.

**Table 2:** Proximate chemical composition of the dietary pumpkin seed meal

Items	PSM
Nutrients (%, on dry matter basis)	
Dry matter	91.90
Crude protein	26.16
Ether extract	26.44
Crude fiber	21.11
PUFA (g/100 g total FA)	
Total PUFA	51.22
n−6 PUFA	48.75
n−3 PUFA	2.47
n−6/n−3 ratio	19.73
Antioxidant compounds	
Polyphenols (mg/g)	25.01
Antioxidant capacity (mM Trolox)	14.80
Flavonoids (mg rutin/g)	8.00

PSM, pumpkin seed meal; PUFA, polyunsaturated fatty acids; FA, fatty acids.

**Table 3:** Effect of dietary pumpkin seed meal on laying hens’ performances

Items	CON	PSM	SEM	p-value
Week 1 to 3				
ADFI (g/hen)	118.75^a^	106.76^b^	1.302	<0.0001
FCR (kg CFs/kg egg)	2.10	2.00	0.034	0.1468
Egg weight (g)	63.65	63.76	0.226	0.1001
Laying rate (%)	89.22	88.92	1.227	0.8436
Week 3 to 6				
ADFI (g/hen)	117.01^a^	111.05^b^	0.867	0.0002
FCR (kg CFs/kg egg)	2.10	2.18	0.039	0.3207
Egg weight (g)	64.23	63.95	0.190	0.2131
Laying rate (%)	87.44	88.64	1.154	0.2302
Overall weeks 1 to 6				
ADFI (g/hen)	117.86^a^	108.96^b^	0.773	<0.0001
FCR (kg CFs/kg egg)	2.10	2.09	0.026	0.8622
Egg weight (g)	63.45	63.91	0.149	0.0851
Laying rate (%)	88.30	87.63	0.852	0.3290
Liveability (%)	100	100	na	na

CON, control diet; PSM, control diet supplemented with 9% pumpkin seed meal; SEM, standard error of the mean; ADFI, average daily feed intake; FCR, feed conversion ratio; CF, compound feed; na, not applicable.

a,b Means within a row with no common superscript differ (p<0.05).

**Table 4:** Influence of dietary pumpkin seed meal on the internal and external quality parameters of the eggs in comparison with control treatment

Items	CON	PSM	SEM	p-value
Egg weight (g)	63.50	63.52	0.208	0.9562
Constituents				
White (g)	37.86	38.55	0.249	0.1710
Yolk (g)	17.00	16.33	0.231	0.1470
Shell (g)	8.64	8.65	0.090	0.9569
External parameters				
Shell thickness (μm)	0.35	0.34	0.004	0.1805
Shell strength (kgF)	3.77	4.09	0.128	0.2183
Yolk colour fan	4.89	5.00	0.068	0.4244
Internal parameters				
White pH	8.55	8.47	0.035	0.1263
Yolk pH	6.30	6.29	0.014	0.4003
Haugh units (HU)	89.73	92.22	1.106	0.5645

CON, control diet; PSM, control diet supplemented with 9% pumpkin seed meal; SEM, standard error of the mean.

**Table 5:** Effect of dietary pumpkin seed meal on fatty acid composition

Item	CON	PSM	SEM	p-value

	------------------------ g/100 g total FA -----------------			
SFA	36.39	36.42	0.456	ns
Myristic C14:0	0.32^a^	0.25^b^	0.014	0.0061
Pentadecanoic C15:0	0.065	0.068	0.005	ns
Palmitic C16:0	25.19	24.27	0.248	ns
Heptadecanoic C17:0	0.11^a^	0.16^b^	0.045	0.0323
Stearic C18:0	10.70	11.67	0.640	ns
MUFA	37.81	35.29	0.763	ns
Palmitoleic C16:1	3.13^a^	2.45^b^	0.150	0.0142
Heptadecenoic C17:1	0.08	0.11	0.009	ns
Oleic C18:1	33.99	32.26	0.640	ns
Erucic C22:1n9	0.11	0.11	0.013	ns
Nervonic C24:1n9	0.37	0.36	0.013	ns
Total PUFA	25.79^a^	28.26^b^	0.615	0.0372
n−6 PUFA	24.58	26.70	0.579	ns
Linoleic C18:2n6	18.37^a^	20.65^b^	0.416	0.0010
Linolenic γ C18:3n6	0.13	0.13	0.006	ns
Eicosadienoic C20:2n6	0.15^a^	0.13^b^	0.006	0.0327
Eicosatrienoic C20:3n6	0.25	0.25	0.021	ns
Arachidonic C20:4n6	4.09	3.93	0.372	ns
n−3 PUFA	1.22^a^	1.66^b^	0.064	0.0013
Linolenic α C18:3n3	0.21^a^	0.33^b^	0.011	0.0022
Eicosatrienoic C20:3n3	0.23	0.25	0.020	ns
Docosapentaenoic C22:5n3	0.08	0.10	0.009	ns
Docosahexaenoic C22:6n3	0.70^a^	0.98^b^	0.052	0.0022
n−6/n−3 ratio	20.36^a^	17.20^b^	0.713	0.0178
Cholesterol (g/100 g dry yolk)	1.865^a^	1.654^b^	0.045	0.0488
Cholesterol (g/100 g egg)	0.260^a^	0.233^b^	0.006	0.0235
Crude fat (%)	28.45	29.10	0.210	ns

CON, control diet; PSM, control diet supplemented with 9% pumpkin seed meal; SEM, standard error of the mean; FA, fatty acids; SFA, saturated fatty acids; MUFA, monounsaturated fatty acids; PUFA, polyunsaturated fatty acids; ns, nonsignificant.

a,b Means within a row with no common superscript differ (p<0.05).
